# Microstructure Abnormalities in Adolescents with Internet Addiction Disorder

**DOI:** 10.1371/journal.pone.0020708

**Published:** 2011-06-03

**Authors:** Kai Yuan, Wei Qin, Guihong Wang, Fang Zeng, Liyan Zhao, Xuejuan Yang, Peng Liu, Jixin Liu, Jinbo Sun, Karen M. von Deneen, Qiyong Gong, Yijun Liu, Jie Tian

**Affiliations:** 1 School of Life Sciences and Technology, Life Sciences Research Center, Xidian University, Xi'an, Shaanxi, China; 2 Department of Applied Mathematics, Xidian University, Xi'an, Shaanxi, China; 3 The 3rd Teaching Hospital, Chengdu University of Traditional Chinese Medicine, Chengdu, Sichuan, China; 4 National Institute on Drug Dependence, Peking University, Beijing, China; 5 Department of Radiology, The Center for Medical Imaging, Huaxi MR Research Center (HMRRC), West China Hospital of Sichuan University, Chengdu, Sichuan, China; 6 Departments of Psychiatry and Neuroscience, McKnight Brain Institute, University of Florida, Gainesville, Florida, United States of America; 7 Institute of Automation, Chinese Academy of Sciences, Beijing, China; University of Illinois at Chicago, United States of America

## Abstract

**Background:**

Recent studies suggest that internet addiction disorder (IAD) is associated with structural abnormalities in brain gray matter. However, few studies have investigated the effects of internet addiction on the microstructural integrity of major neuronal fiber pathways, and almost no studies have assessed the microstructural changes with the duration of internet addiction.

**Methodology/Principal Findings:**

We investigated the morphology of the brain in adolescents with IAD (N = 18) using an optimized voxel-based morphometry (VBM) technique, and studied the white matter fractional anisotropy (FA) changes using the diffusion tensor imaging (DTI) method, linking these brain structural measures to the duration of IAD. We provided evidences demonstrating the multiple structural changes of the brain in IAD subjects. VBM results indicated the decreased gray matter volume in the bilateral dorsolateral prefrontal cortex (DLPFC), the supplementary motor area (SMA), the orbitofrontal cortex (OFC), the cerebellum and the left rostral ACC (rACC). DTI analysis revealed the enhanced FA value of the left posterior limb of the internal capsule (PLIC) and reduced FA value in the white matter within the right parahippocampal gyrus (PHG). Gray matter volumes of the DLPFC, rACC, SMA, and white matter FA changes of the PLIC were significantly correlated with the duration of internet addiction in the adolescents with IAD.

**Conclusions:**

Our results suggested that long-term internet addiction would result in brain structural alterations, which probably contributed to chronic dysfunction in subjects with IAD. The current study may shed further light on the potential brain effects of IAD.

## Introduction

As an important period between childhood and adulthood, adolescence is encompassed by alterations in physical, psychological, and social development [1]. During this developmental stage, more time is spent with peers and adults to face the variant social environment where more conflicts arise [2]. The presence of relatively immature cognitive control [3]–[7], makes this period a time of vulnerability and adjustment [8] and may lead to a higher incidence of affective disorders and addiction among adolescents [8]–[10]. As one of the common mental health problems amongst Chinese adolescents, internet addiction disorder (IAD) is currently becoming more and more serious [11].

The use of the internet has expanded incredibly across the world over the last few years. The internet provides remote access to others and abundant information in all areas of interest. However, maladaptive use of the internet has resulted in impairment of the individual's psychological well-being, academic failure and reduced work performance [12]–[18]. While not yet officially codified within a psychopathological framework, IAD is growing in prevalence and has attracted the attention of psychiatrists, educators, and the public. The relatively immature cognitive control of the adolescents puts them at a high risk of contracting IAD. Some adolescents cannot control their impulsive use of the internet for novelty seeking and finally become addicted to the internet. Data from the China Youth Internet Association (announcement on February 2, 2010) demonstrated that the incidence rate of internet addiction among Chinese urban youths is about 14%. It is worth noting that the total number is 24 million (http://www.zqwx.youth.cn/).

Numerous IAD studies have been carried out across the world and obtained some interesting findings [11], [15], [19]–[22]. Ko et al. [19] identified the neural substrates of online gaming addiction via evaluation of the brain areas associated with the cue-induced gaming urge, which consisted of the right orbitofrontal cortex (OFC), right nucleus accumbens (NAc), bilateral anterior cingulated cortex (ACC), medial frontal cortex, right dorsolateral prefrontal cortex (DLPFC), and right caudate nucleus. Due to the similarity of the cue-induced craving in substance dependence, they suggested that the gaming urge/craving in online gaming addiction and craving in substance dependence might share the same neurobiological mechanisms. Cao et al. [11] found that Chinese adolescents with IAD exhibited more impulsivity than controls. Recently, Dong et al. [20] investigated response inhibition in people with IAD by recording event-related brain potentials during a Go/NoGo task and showed that the IAD group exhibited a lower NoGo-N2 amplitude, higher NoGo-P3 amplitude, and longer NoGo-P3 peak latency than the normal group. They suggested that the IAD subjects had lower activation in the conflict detection stage than the normal group; thus, they had to engage in more cognitive endeavors to complete the inhibition task in the late stage. In addition, the IAD subjects showed less efficiency in information processing and lower cognitive control [20]. Some researchers also detected gray matter density deficits [21] and resting-state abnormalities [22] in IAD subjects, such as lower gray matter density in the left ACC, left posterior cingulate cortex (PCC), left insula, and left lingual gyrus and increased regional homogeneity (ReHo) in the right cingulate gyrus, bilateral parahippocampus and some other brain regions.

Unfortunately, there is currently no standardized treatment for IAD. Clinics in China have implemented regimented timetables, strict discipline and electric shock treatment, which gained notoriety for these treatment approaches [13]. Developing effective methods for intervention and treatment of IAD will require first establishing a clear understanding of the mechanisms underlying this disease. However, few studies reported the abnormalities of white matter in the adolescents with IAD. Knowledge of the brain abnormalities of gray matter and white matter and association between these abnormalities and cognitive functions in IAD subjects is helpful to identify possible pharmacotherapies to treat this disorder. Advances in neuroimaging techniques provide us with ideal methods to investigate these issues [23]–[27]. In this study, we investigated the morphology of the brain in adolescents with IAD using an optimized voxel-based morphometry (VBM) technique and studied white matter fractional anisotropy (FA) changes using the diffusion tensor imaging (DTI) method, and linked these brain structural measures to the duration of IAD. We can draw a conclusion from previous IAD studies that the IAD subjects showed impaired cognitive control, and we hypothesized that long-term internet addiction would result in brain structural alterations and these structural abnormalities were associated with functional impairments in cognitive control in IAD subjects [15], [16], [20], [28]. Furthermore, the structural abnormalities of certain brain regions would correlate with the duration of IAD.

## Materials and Methods

All research procedures were approved by the West China Hospital Subcommittee on Human Studies and were conducted in accordance with the Declaration of Helsinki.

### 2.1 Subjects

According to the modified Young Diagnostic Questionnaire for Internet addiction (YDQ) criteria by Beard and Wolf [16], [29], eighteen freshman and sophomore students with IAD (12 males, mean age  = 19.4±3.1 years, education 13.4±2.5 years) were engaged in our study. The YDQ criteria [16] consisted of the following eight “yes” or “no” questions which were: (1) Do you feel absorbed in the Internet (remember previous online activity or the desired next online session)? (2) Do you feel satisfied with Internet use if you increase your amount of online time? (3) Have you failed to control, reduce, or quit Internet use repeatedly? (4) Do you feel nervous, temperamental, depressed, or sensitive when trying to reduce or quit Internet use? (5) Do you stay online longer than originally intended? (6) Have you taken the risk of losing a significant relationship, job, educational or career opportunity because of the Internet? (7) Have you lied to your family members, therapist, or others to hide the truth of your involvement with the Internet? (8) Do you use the Internet as a way of escaping from problems or of relieving an anxious mood (e.g., feelings of helplessness, guilty, anxiety, or depression)? All of the eight questions were translated into Chinese. Young asserted that five or more “yes” responses to the eight questions indicated an internet-dependent user [16]. Later on, Beard and Wolf modified the YDQ criteria [29], and respondents who answered “yes” to questions 1 through 5 and at least to any one of the remaining three questions were classified as suffering from internet addiction, which was used for screening the subjects in the present study. The addiction was a gradual process, so we investigated whether or not there were any linear changes in the brain structure. The duration of the disease was estimated via a retrospective diagnosis. We asked the subjects to recall their life-style when they were initially addicted to the internet. To guarantee that they were suffering from internet addiction, we retested them with the YDQ criteria modified by Beard and Wolf. We also confirmed the reliability of the self-reports from the IAD subjects by talking with their parents via telephone. The IAD subjects spent 10.2±2.6 hours per day on online gaming. The days of internet use per week was 6.3±0.5. We also verified this information from the roommates and classmates of the IAD subjects that they often insisted being on the internet late at night, disrupting others' lives despite the consequences. Eighteen age- and gender-matched (*p*>0.01) healthy controls (12 males, mean age  = 19.5±2.8 years, education 13.3±2.0 years) with no personal or family history of psychiatric disorders also participated in our study. According to a previous IAD study [19], we chose healthy controls who spent less than 2 hours per day on the internet. The healthy controls were also tested with the YDQ criteria modified by Beard and Wolf to ensure they were not suffering from IAD. All recruited participants screened were native Chinese speakers, never used illegal substances, and were right-handed. Prior to magnetic resonance imaging (MRI) scanning, urine drug screening was performed on all subjects to exclude substance abuse. Exclusion criteria for both groups were (1) existence of a neurological disorder; (2) alcohol, nicotine or drug abuse; (3) pregnancy or menstrual period in women; and (4) any physical illness such as a brain tumor, hepatitis, or epilepsy as assessed according to clinical evaluations and medical records. Furthermore, the Self-Rating Anxiety Scale (SAS) and the Self-Rating Depression Scale (SDS) were used to evaluate the emotional states of all participants on the day of the scans. All patients and healthy controls gave written informed consent. More detailed demographic information was given in Table 1.

**Table 1:** Subject demographics for internet addiction disorder (IAD) and control groups.

Items	IADN = 18	ControlN = 18	*P* value
Age (years)	19.4±3.1	19.5±2.8	>0.05
Education (years)	13.4±2.5	13.3±2.0	>0.05
Duration of internet addiction (months)	34.8±8.5	N/A	N/A
Hours of internet use (/day)	10.2±2.6	0.8±0.4	**
Days of internet use(/week)	6.3±0.5	1.6±0.8	**
Self-Rating Anxiety Scale	28.8±5.3	27.4±4.8	>0.05
Self-Rating Depression Scale	43.2±8.9	28.5±5.2	*

*: *p*<0.05;

**: *p*<0.005.

### 2.2 Brain Imaging Methodology and Data Analysis

#### 2.2.1 Scanning parameters

Imaging data was performed on a 3T Siemens scanner (Allegra; Siemens Medical System) at the Huaxi MR Research Center, West China Hospital of Sichuan University, Chengdu, China. A standard birdcage head coil was used, along with restraining foam pads to minimize head motion and to diminish scanner noise. Image sequences were acquired by means of diffusion weighted imaging with single-shot echo planar imaging in alignment with the anterior–posterior commissural plane. Diffusion tensor images were acquired with 2 averages. The diffusion sensitizing gradients were applied along 30 non-linear directions (b = 1000 s/mm^2^) together with an acquisition without diffusion weighting (b = 0 s/mm^2^). The imaging parameters were 45 continuous axial slices with a slice thickness of 3 mm and no gap, field of view  = 240×240 mm^2^, repetition time/echo time  = 6800/93 ms, acquisition matrix  = 128×128. In addition, the axial 3D T1-weighted images were obtained with a spoiled gradient recall sequence and the following parameters: TR = 1900 ms; TE = 2.26 ms; flip angle  = 9^0^; in-plane matrix resolution  = 256×256; slices  = 176; field of view  = 256 mm; voxel size  = 1×1×1 mm.

#### 2.2.2 VBM

Structural data was processed with an FSL-VBM protocol [30], [31] with FSL 4.1 software [32]. First, all T1 images were brain-extracted using the brain extracting tool (BET) [33]. Next, tissue-type segmentation was carried out using FMRIB's automated segmentation tool (FAST) V4.1 [34]. The resulting gray matter partial volume images were then aligned to MNI152 standard space using the FMRIB's linear image registration tool (FLIRT) [35], [36], followed optionally by nonlinear registration using the FMRIB's nonlinear image registration tool (FNIRT) [37], [38], which uses a b-spline representation of the registration warp field [39]. The resulting images were averaged to create a study-specific template, to which the native gray matter images were then non-linearly re-registered. The optimized protocol introduced a modulation for the contraction/enlargement due to the nonlinear component of the transformation: each voxel of the registered gray matter image was divided by the Jacobian of the warp field. Finally, in order to choose the best smoothing kernel, all 32 modulated, normalized gray matter volume images were smoothed with isotropic Gaussian kernels increasing in size (sigma  = 2.5, 3, 3.5, and 4 mm, corresponding to a 6, 7, 8, and 9.2 mm FWHM respectively). Regional changes in gray matter were assessed using permutation-based non-parametric testing with 5000 random permutations [40]. Analysis of covariance (ANCOVA) was employed with age, gender effects and total intracranial volume as covariates. Total intracranial volume was calculated as the sum of gray matter, white matter, and cerebrospinal fluid volumes from FSL BET segmentations. Recently, Dong et al. found that depression and anxiety scores were significantly higher after the addiction compared to before the addiction in some college students, and they suggested that these were outcomes of IAD, hence SAS and SDS were not included as confounds [41]. Correction for multiple comparisons was carried out using a cluster-based thresholding method, with an initial cluster forming a threshold at t = 2.0. Results were considered significant for *p*<0.05. For the regions where IAD subjects showed significantly different gray matter volume from the controls, the gray matter volumes of these areas were extracted, averaged and regressed against the duration of internet addiction.

#### 2.2.3 DTI

We calculated the FA value for each voxel, which reflected the degree of diffusion anisotropy within a voxel (range 0–1, where smaller values indicated more isotropic diffusion and less coherence and large values indicated directional dependence of Brownian motion due to white matter tracts) [42]. FDT software in FSL 4.1 was used for FA calculation [32]. First of all, correction for eddy-currents and head motion was done by means of affine registration on the first no-diffusion weighted volume of each subject. FA images were created by fitting the diffusion tensor to the raw diffusion data after brain extraction using BET [33]. Then, a voxel-wise statistical analysis of the FA data was carried out using the tract-based spatial statistics (TBSS) V1.2 part of FSL [43], [44]. FA imges from all of the subjects (IAD subjects and healthy controls) were realigned into an FMRIB58_FA standard-space image by FNIRT [37], [38] using a b-spline representation of the registration warp field [39]. The mean FA image was then created and thinned to create a mean FA skeleton (threshold of 0.2) representing the centers of all of the tracts common to the group. Each subject's aligned FA data was then projected back onto this skeleton. White matter FA value changes were assessed using permutation-based non-parametric testing [40] with 5000 random permutations. ANCOVA was employed with age and gender effects as covariates. Correction for multiple comparisons was carried out using a cluster-based thresholding method, with an initial cluster forming threshold of t = 2.0. Results were considered significant for *p*<0.05. For the clusters where internet addiction subjects showed significantly different FA values from the controls, the FA of these brain regions were extracted, averaged and regressed against the duration of internet addiction.

#### 2.2.4 Interaction between gray matter and white matter abnormalities

To investigate the interactions between gray matter changes and white matter alterations, a correlation analysis was performed between abnormal gray matter volumes and white matter FA values in the IAD group.

## Results

### 3.1 VBM results

**Figure 1:**
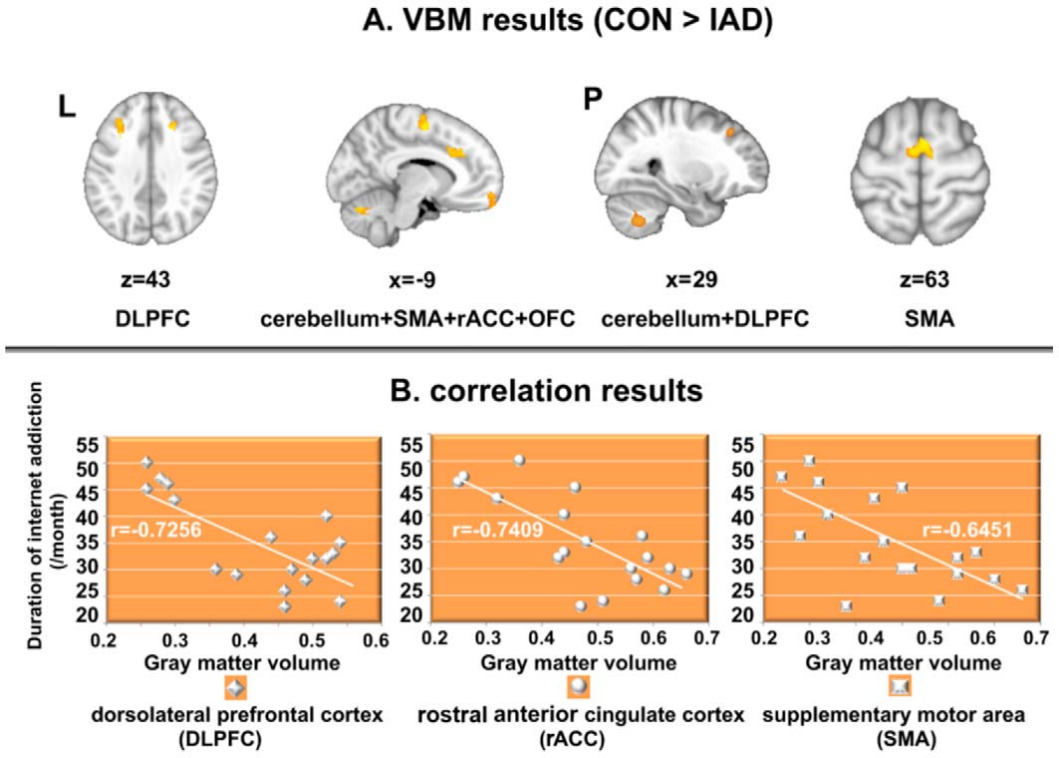
VBM results. A. Reduced gray matter volume in IAD subjects, (1-*p*) corrected *p*-value images. The background image is the standard MNI152_T1_1mm_brain template in FSL. B. The gray matter volumes of the DLPFC, rACC and SMA were negatively correlated with the duration of internet addiction.

Regional gray matter volume changes were assessed non-parametrically using optimized VBM. Correction for multiple comparisons was carried out using a cluster-based thresholding method. VBM comparison between IAD subjects and matched healthy controls indicated decreased gray matter volume in several clusters, i.e. the bilateral DLPFC, the supplementary motor area (SMA), the OFC, the cerebellum and the left rostral ACC (rACC), after controlling for potential confounding variables including age, gender effects and total intracranial volume. Gray matter volumes of the right DLPFC, the left rACC and the right SMA showed a negative correlation with months of internet addiction (r1 = −0.7256, *p1* < 0.005; r2 = −0.7409, *p2* < 0.005; r3 = −0.6451, *p3* < 0.005). No brain regions showed higher gray matter volume than healthy controls as shown in Figure 1 and Table 2.

**Table 2:** Regions that showed abnormal gray matter volume and white matter FA (fractional anisotropy) between subjects with internet addiction disorder (IAD) and healthy controls (*p*<0.05 corrected).

Regions	Side	MNI Coordinates			*P*-valuescorrected
		x	y	z	
VBM results (CON>IAD)					
DLPFC(BA46)	L	−32	26	36	0.013
DLPFC(BA46)	R	27	29	37	0.024
rACC(BA32)	L	−9	25	26	0.026
OFC(BA11)	L	−9	58	−15	0.027
OFC(BA11)	R	17	61	−5	0.016
SMA(BA6)	L	−4	−8	63	0.031
SMA(BA6)	R	6	−4	63	0.023
Cerebellum	L	−27	−61	−33	0.022
Cerebellum	R	28	−56	−48	0.015
DTI results					
PHG(CON>IAD)	R	22	−38	−1	0.023
PLIC(CON<IAD)	L	−13	−7	−3	0.014

DLPFC, dorsolateral prefrontal cortex; rACC, rostral anterior cingulate cortex; OFC, orbitofrontal cortex; SMA, supplementary motor area; PHG, parahippocampal gyrus; PLIC, posterior limb of the internal capsule; MNI, Montreal Neurological Institute; VBM, voxel-based morphometry; DTI, diffusion tensor imaging; CON, control; IAD; internet addiction disorder; BA, Brodmann area; L, left; and R, right.

### 3.2 DTI results

**Figure 2:**
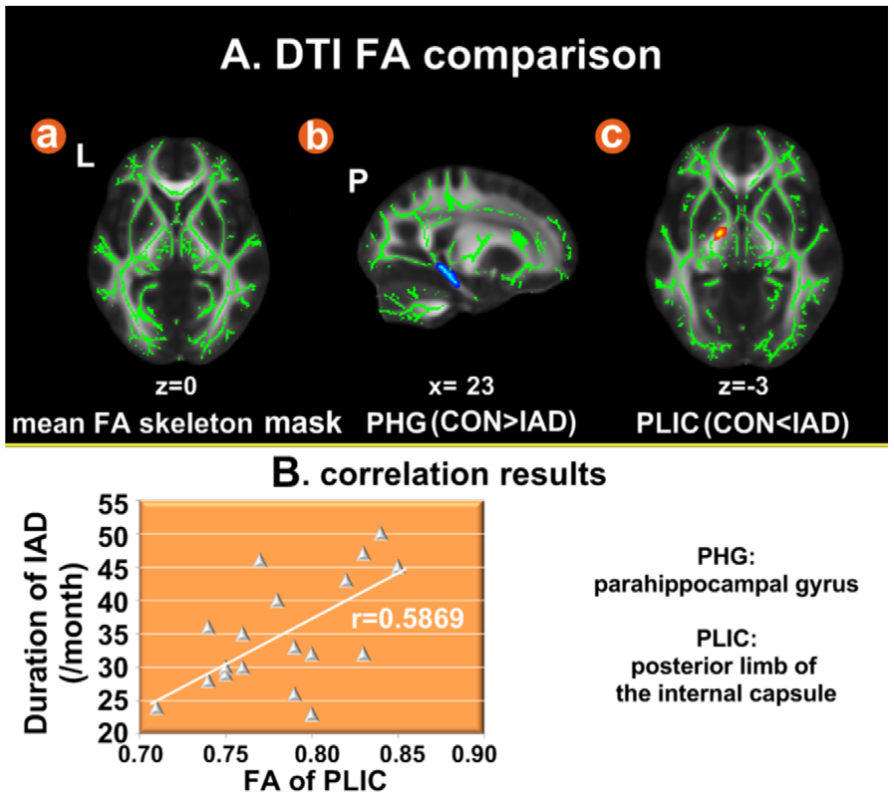
DTI results. A. White matter structures showing abnormal FA in IAD subjects, (1-*p*) corrected *p*-value images. The background image is the standard FMRIB58_FA_1mm template in FSL. Red-Yellow voxels represent regions in which FA was decreased significantly in IAD relative to healthy controls. Blue-Light Blue voxels represent increased FA in IAD. B. The FA of the PLIC was positively correlated with the duration of internet addiction.

With regards to DTI data analysis, correction for multiple comparisons was carried out using the cluster-based thresholding method. Our TBSS results revealed enhanced FA value (IAD: 0.78±0.04; control: 0.56±0.02) of the left posterior limb of the internal capsule (PLIC) in IAD subjects compared to healthy controls and reduced FA value (IAD: 0.31±0.04 ; control: 0.48±0.03) in the white matter within the right parahippocampal gyrus (PHG) as shown in Figure 2 and Table 2. Furthermore, the FA tended to correlate positively with the duration of internet addiction in the left PLIC (r = 0.5869, *p* < 0.05), whereas no significant correlation was observed between the FA value of the right PHG and duration of internet addiction.

### 3.3 Interaction between gray matter and white matter abnormalities

The interaction analysis between gray matter volumes and white matter FA values in IAD group revealed that there were no significant correlations between these two measures.

## Discussion

IAD resulted in impaired individual psychological well-being, academic failure and reduced work performance among adolescents [12]–[18]. However, there is currently no standardized treatment for IAD. Developing effective methods for intervention and treatment of IAD will require first establishing a clear understanding of the mechanisms. Awareness of the brain structural abnormalities in IAD is critical for identifying possible pharmacotherapies to treat this disorder. In the present study, we discovered gray matter volume changes and white matter FA changes in adolescents with IAD. We also revealed the association between these structural abnormalities and duration of internet addiction. We suggested that IAD resulted in brain structural changes in the adolescents and these structural abnormalities were probably associated with functional impairments in cognitive control.

### 4.1 VBM results

Consistent with a previous VBM study [21], we found no brain regions showing increased gray matter volume in internet addiction subjects. Regional gray matter volume comparison indicated atrophy within several clusters for the entire group of internet addicts (*p* < 0.05, corrected), which were the bilateral DLPFC, SMA, cerebellum, OFC and the left rACC (as shown in Figure 1). Moreover, atrophy of the right DLPFC, the left rACC and the right SMA was negatively correlated with the duration of internet addiction, which Zhou et al. failed to detect [21]. These results demonstrated that as internet addiction persisted, brain atrophy of the DLPFC, rACC and SMA was more serious. Some results of brain atrophy in our study were different from previous findings [21], which may be due to the different data processing methods. In the present study, the possible confounding effects of age, gender and the whole brain volume were included as covariates, which the previous study failed to consider. The different processing methods possibly gave rise to the different findings.

According to previous drug addiction studies, long-term substance abuse [45], [46] and internet addiction [11], [20] will lead to impaired cognitive control. Cognitive control can be conceptualized as the capacity to suppress prepotent but incorrect responses and the ability to filter out irrelevant information within a stimulus set and allow appropriate actions to meet complicated task demands and adaptation to changing environments [47]. Numerous functional brain imaging studies have revealed that the DLPFC and rACC were centrally involved in cognitive control [48], [49]. Different neurocognitive studies have revealed that cognitive control is related to a specific cortico–subcortical circuit, including the rACC and the DLPFC [50], [51]. According to a prominent conflict-monitoring hypothesis [47], [52], the occurrence of response conflict is signaled by the rACC, leading to recruitment of the DLPFC for more cognitive control for subsequent performance. This important role of the DLPFC has been identified in neuroscience research with top-down regulatory processes of cognitive control [53]. Recent neuroimaging studies have also disclosed deactivation of the rACC in a GO/NOGO task in heroin-dependent individuals [54], [55] and cocaine users [45], indicating the critical role of the rACC in cognitive control [46].

The OFC is also thought to contribute to cognitive control of goal-directed behavior through the assessment of the motivational significance of stimuli and the selection of behavior to obtain desired outcomes [56]. The OFC has extensive connections with the striatum and limbic regions (such as the amygdala). As a result, the OFC is well situated to integrate the activity of several limbic and subcortical areas associated with motivational behavior and reward processing [57]. Some animal studies have demonstrated that damage to both of the OFC and the rat prelimbic cortex (the functional homologue of the human DLPFC) impaired the acquisition and modification of behavior guided by contingencies between responses and outcomes, indicating that these regions may be crucial for the cognitive control of goal-directed behavior [56], [58].

The SMA is critical for selection of appropriate behavior, whether selecting to execute an appropriate response or selecting to inhibit an inappropriate response [59]. Some researchers detected that both simple and complex GO/NOGO tasks were involved in the SMA and they revealed the important role of the SMA in mediating cognitive control [46], [60].

Several anatomical, physiological, and functional imaging studies suggest that the cerebellum contributes to higher-order cognitive functions [61]–[64], with discrete lesions to the cerebellum resulting in impairment of executive functions and working memory, even in personality changes such as disinhibited and inappropriate behavior.

Our results (Figure 1) of the reduced gray matter volume in the DLPFC, rACC, OFC, SMA and cerebellum may be, at least in part, associated with cognitive control and goal-directed behavior dysfunctions in internet addiction [15], [19], [20], [28], which may explain fundamental symptoms of internet addiction.

### 4.2 DTI results

We calculated the FA value in each white matter voxel for each subject, which quantified the strength of directionality of the local tract microstructure. The whole brain voxel-wise comparison over the white matter skeleton using permutation testing and stringent statistical thresholding indicated that IAD subjects had lower FA values in a cluster within the right PHG (*p* < 0.05, corrected). On the other hand, searching for increased FA in IAD subjects showed that IAD subjects had higher FA values in a cluster within the left PLIC (*p* < 0.05, corrected). Moreover, the FA value of the left PLIC was positively correlated with the duration of internet addiction (Figure 2).

The PHG is a brain region that surrounds the hippocampus and plays an important role in memory encoding and retrieval [65], [66]. The PHG provides the major poly-sensory input to the hippocampus through the entorhinal connections and is the recipient of different combinations of sensory information [67], [68], which are involved in cognition and emotional regulation [69]. Recently, some researchers suggested that the right PHG contributes to the formation and maintenance of bound information in working memory [70]. Working memory is devoted to the temporary storage and on-line manipulation of information and is critical for cognitive control [71]. The finding that the lower FA value of the PHG in IAD subjects demonstrated that abnormal white matter properties maybe the structural basis of functional deficits of working memory in IAD subjects [19]. Recently, Liu et al. [72] reported increased ReHo in the bilateral PHG in IAD college students compared with the controls and suggested this result reflected the functional change in the brain, possibly relating to the reward pathways. Evidently, more work is needed to understand the accurate role of the PHG in IAD.

Anatomically, the internal capsule is an area of white matter in the brain that separates the caudate nucleus and the thalamus from the lenticular nucleus, which contains both ascending and descending axons. In addition to corticospinal and corticopontine fibers, the internal capsule contains thalamocortical and corticofugal fibers [73], [74]. The posterior limb of the internal capsule contains corticospinal fibers, sensory fibers (including the medial lemniscus and the anterolateral system) from the body and a few corticobulbar fibers [73]–[76]. The primary motor cortex sends its axons through the posterior limb of the internal capsule and plays important roles in finger movement and motor imagery [77], [78]. The possible reason for the FA values in internal capsule enhancement was that the IAD subjects spent more time playing computer games and the repetitive motor actions such as clicking the mouse and keyboard typing changed the structure of the internal capsule. As the findings that training modified the brain structure in other studies [79]–[81], these long-term training probably changed the white matter structure of the PLIC. Information transmission between frontal and subcortical brain regions modulated higher cognitive functioning and human behaviors [82], [83], which relied on the white matter fiber tracts passing through the internal capsule [83], [84]. Structural abnormalities in the internal capsule could consequently interfere with the cognitive function and impair executive and memory functions [85]. The abnormal FA value of the left PLIC may influence the sensory information transfer and processing, and finally lead to impairments in cognitive control [86], [87]. Moreover, being addicted to the internet can cause physical discomfort or medical problems such as: carpal tunnel syndrome, dry eyes, backaches, and severe headaches [88]–[90]. The abnormal FA value of the left PLIC may explain the carpal tunnel syndrome in IAD subjects, which needs to be verified with a more sophisticated design in the future.

### 4.3 Interaction between gray matter and white matter abnormalities

We have investigated the relationship between gray matter and white matter alterations. Unfortunately, there were no significant correlations between these two measures. This phenomenon suggested that the morphological changes of IAD on the brain's gray matter and white matter were not significantly linear correlated. There existed the possibility that the gray matter abnormalities linked the white matter alterations in some other way. However, our findings demonstrated that the structure characteristics of gray matter and white matter were abnormal in the adolescents with IAD.

There are some limitations of the current study. First of all, while our results have indicated that the gray matter and white matter changes may be the consequence of excessive internet use or IAD, we can't exclude another possibility which addresses the structural difference between the normal controls and IAD that may be the cause for the over-use of the internet. The abnormal characteristics of these cognitive control-related brain regions in some adolescents make them relatively immature and allow them to be easily internet dependent. The cause and consequence issues should be investigated by a more comprehensive experimental design in the future study. However, we suggested that the findings in the present study were the consequence of IAD. Secondly, with regard to the relationship between the structural changes and duration of IAD, the months of IAD is a gross characterization by the recollection of the IAD subjects. We asked the subjects to recall their life-style when they were initially addicted to the internet. To guarantee that they were suffering from internet addiction, we retested them with the YDQ criteria modified by Beard and Wolf. We also confirmed the reliability of the self-reports from the IAD subjects by talking with their parents over the telephone. The brain structural changes in accordance with the addiction process may be more crucial in understanding the disease, hence the correlation between duration and the brain structural measures was carried out. These correlations suggested that cumulative effects were found in the reduced gray matter volume of the right DLPFC, the right SMA, the left rACC and increased white matter FA in the left PLIC. Finally, although we suggested that the structural abnormalities of the gray matter volume and white matter FA were associated with functional impairments in cognitive control in IAD, the biggest limitation of the present study is the lack of quantitative indication of the deficits in cognitive control in these adolescents with IAD. Although the relationships between these structural abnormalities and duration of internet addiction were verified in our current study, fully characterizing the nature of the underlying structural abnormalities in IAD is still needed to be researched in greater detail in the future, which is critical in understanding the impact of IAD on long-term functioning. In the future, we will integrate these structural findings with behavioral performances of cognitive tasks in subjects with IAD. Overall, the FA changes and gray matter volume changes as shown in the present study indicated an alteration in the brain on a microstructural level, which enhanced our understanding of IAD.

### Conclusion

We provided evidences indicating that IAD subjects had multiple structural changes in the brain. The gray matter atrophy and white matter FA changes of some brain regions were significantly correlated with the duration of internet addiction. These results may be interpreted, at least partially, as the functional impairment of cognitive control in IAD. The prefrontal cortex abnormalities were consistent with previous substance abuse studies [23], [48], [80], [81], hence we suggested that there may exist partially overlapping mechanisms in IAD and substance use. We hoped that our results will enhance our understanding of IAD and aid in improving the diagnosis and prevention of IAD.
